# The effects of maternal iron deficiency on infant fibroblast growth factor-23 and mineral metabolism

**DOI:** 10.1016/j.bone.2015.10.003

**Published:** 2016-02

**Authors:** V.S. Braithwaite, A. Prentice, M.K. Darboe, A.M. Prentice, S.E. Moore

**Affiliations:** a MRC Human Nutrition Research, Elsie Widdowson Laboratory, Fulbourn Road, Cambridge CB1 9NL, UK and MRC Unit, The Gambia; b MRC International Nutrition Group at London School of Hygiene & Tropical Medicine, Keppel St, London WC1E 7HT, UK and MRC Unit, The Gambia

**Keywords:** Iron status in pregnancy, FGF23, Phosphate, Bone health, Africa, Early life

## Abstract

Fibroblast growth factor-23 (FGF23), a phosphate(Phos)-regulating hormone, is abnormally elevated in hypophosphataemic syndromes and an elevated FGF23 is a predictor of mortality in kidney disease. Recent findings suggest iron deficiency as a potential mediator of FGF23 expression and murine studies have shown *in utero* effects of maternal iron deficiency on offspring FGF23 and phosphate metabolism.

Our aim was to investigate the impact of maternal iron status on infant FGF23 and mineral metabolites over the first 2 years of life. Infants born to mothers with normal (**NI***n* = *25*,) and low (**LI***n* = *25*) iron status during pregnancy, from a mother-infant trial (ISRCTN49285450) in rural Gambia, West Africa, had blood and plasma samples analysed at 12, 24, 52, 78 and 104 weeks (wk) of age.

Circulating intact-FGF23 (I-FGF23), Phos, total alkaline phosphatase (TALP) and haemoglobin (Hb) decreased and estimated glomerular filtration rate increased over time [all *P* ≤ 0.0001)]. C-terminal-FGF23 (C-FGF23) and TALP were significantly higher in **LI** compared with **NI,** from 52 wk for C-FGF23 [Beta coefficient (SE) 18.1 (0.04) %, *P* = 0.04] and from 24 wk for TALP [44.7 (29.6) U/L, *P* = 0.04]. Infant Hb was the strongest negative predictor of C-FGF23 concentration [− 21% (4%) RU/mL, P ≤ 0.0001], Phos was the strongest positive predictor of I-FGF23 [32.0(3.9) pg/mL, *P* ≤ 0.0001] and I-FGF23 did not predict C-FGF23 over time [− 0.5% (0.5%), *P* = 0.3].

In conclusion, this study suggests that poor maternal iron status is associated with a higher infant C-FGF23 and TALP but similar I-FGF23 concentrations in infants and young children. These findings further highlight the likely public health importance of preventing iron deficiency during pregnancy. Whether or not children who are born to iron deficient mothers have persistently high concentrations of these metabolites and are more likely to be at risk of impaired bone development and pre-disposed to rickets requires further research.

## Highlights

• We measured bone metabolites in infants born to mothers with low (LI) and normal iron (NI) status.
• Infants in the LI group had higher plasma C-terminal FGF23 and TALP compared with the NI group.
• Infant Intact FGF23 did not differ by group.
• Maternal iron status in pregnancy may influence infant bone metabolism.

## 1 Introduction

An elevated concentration of circulating fibroblast growth factor-23 (FGF23) has been shown to be involved in an ever growing set of diseases since its discovery as the cause of certain forms of bone mineralisation disorders such as hereditary hypophosphataemic rickets [1]. These include renal disease and arterial calcification, and an elevated FGF23 concentration is a predictor of mortality in these patients [2]. FGF23, mainly expressed in osteocytes, is primarily a regulator of phosphate (Phos) homeostasis, acting predominantly at the kidney to increase Phos excretion by regulating sodium-phosphate co-transporters, but it also regulates CYP27B1 and CYP24A1 enzyme activity and thus has effects on vitamin D metabolism [3], [4].

The regulatory mechanisms of *FGF23* gene expression and subsequent protein processing in the osteocyte are unclear but a role for iron in FGF23 regulation has been identified. A growing body of evidence has shown inverse associations between markers of iron status with FGF23 concentration in animals [5], [6] and human studies in Africa [7], [8], [9], [10], North America [11] and Poland [12]. A murine study has indicated that maternal iron status during pregnancy influences FGF23 and Phos regulation in infancy [6]. The study showed that pups born to iron deficient dams had significantly higher serum C-terminal and Intact- FGF23 concentrations and correspondingly lower Phos and 1,25-dihydroxyvitamin D (1,25(OH)_2_D) concentrations compared with pups born to iron replete dams [6]. The impact of prenatal iron status on FGF23 and Phos regulation and the corresponding impact on bone health have not yet been investigated in humans. This may be especially relevant in populations with high rates of iron deficiency.

The aim of the current study was to investigate the influence of maternal iron status on circulating FGF23 and mineral homeostasis during early life in rural Gambians; a population in which iron deficiency is common. To this end, the changes in circulating FGF23 and markers of iron and Phos metabolism from 12 weeks (wk) to 2 years of age were measured longitudinally in Gambian children born to mothers with normal and low iron status during pregnancy.

## 2 Materials & methods

### 2.1 Participants

The West Kiang province of The Gambia, West Africa, is a subsistence agricultural region with a high prevalence of iron deficiency anaemia particularly among children and in pregnant women [13]. In addition, elevated FGF23 concentrations have been detected in children with rickets-like bone deformities in this area [14]. Participants were recruited from the MRC (Medical Research Council) Keneba field station, as part of the Early Nutrition and Immune Development trial of pregnant women and their infants (ISRCTN49285450) in which women were randomised to 4 intervention groups from < 20 wk gestation [15]. All groups received iron and folate (as per standard clinical care) with or without additional multiple micronutrients and or protein energy throughout pregnancy [15]. At 6 to 18 months of age, the corresponding children were then further randomised to a lipid-based nutritional supplement, with or without additional multiple micronutrients forming a total of 8 different supplement groups. Ethical approval was given by The Gambian Government/MRC Unit Joint Ethics Committee and written informed consent was obtained from participants or their parents/guardians. The study was conducted in accordance with the Declaration of Helsinki.

### 2.2 Sample collection and biochemical analysis

An overnight-fasted venous blood sample was collected in mothers at “booking” (~ 14 wk of pregnancy) and at 20 and 30 wk of pregnancy and in infants at 12, 24, 52, 78 and 104 wk of age. Blood samples were collected into lithium heparin (LiHep) and EDTA-coated tubes. Haemoglobin (Hb) was measured in maternal and infant whole blood samples by haemoglobinometer (Medonic CA 530 Haematology Analyser, Boule Medical AB, Stockholm, Sweden). Blood samples were then separated by centrifugation after which the plasma was frozen at − 70 °C until analysis. Maternal LiHep samples and infant samples at 24 wk were analysed for ferritin (Ferr), iron (Fe), unbound iron binding capacity (UIBC) and transferrin (Tf). Soluble transferrin receptor (sTfR) and c-reactive protein (CRP) were measured in maternal samples only (Cobas, Integra 400 Plus, Roche Diagnostics, Indiana, USA) at all time points. Infant LiHep and EDTA plasma samples were transported on dry ice to MRC Human Nutrition Research (HNR), Cambridge, UK, for analysis of calcium (Ca), phosphate (Phos), albumin (Alb), magnesium (Mg), total alkaline phosphatase (TALP), and cystatin C (Cys C) (Kone Analyser 20i, Finland) and C-terminal and intact- fibroblast growth factor-23 (C-FGF23 & I-FGF23; Immutopics, CA, USA and Kainos, Japan respectively) at all time points. Assay accuracy and precision were monitored across the working range of the assays using kit controls supplied by the manufacturer. In addition, an aliquot of a pooled plasma sample was assayed in each batch to monitor possible drift over time. Intra and inter- assay coefficients of variation were < 12% for I-FGF23, < 7% for Cys C, < 4% for C-FGF23, and < 2% for the remaining analytes.

### 2.3 Anthropometry and characteristics

Infant length, weight and head-circumference were measured at all time points, using standard protocols and with regularly calibrated equipment. Weight-for-age, length-for-age, weight-for-length and head-circumference-for-age sex specific Z-scores were calculated using WHO reference data [16].

All infants were breast-fed into the second year of life. The infants were exclusively breastfed until a mean age of 5.3 months. This timing did not differ significantly by group 5.3 (1.3) vs. 5.4 (1.1) months (P = 0.8) (Moore S.E, Personal Communication).

### 2.4 Sample selection for analysis

To detect a difference of ~ 67 RU/mL (or 0.75 of a standard deviation based on existing Gambian data (not published)) in plasma C-FGF23 between two groups at β = 80% power α = 0.05 it was estimated that each group needed 25 infants. Samples were selected on the basis of maternal iron status at 20 wk gestation (n = 400). All maternal samples with a CRP < 5 mg/L and a measurement of Ferr and sTfR at 20 wk together with infants who had sufficient EDTA plasma at all 5 time points were eligible for selection (*n* = 85). Women were then ranked by sTfR/logFerr index and grouped into those with an sTfR (mg/L)/logFerr (μg/L) > 2.0 for low iron status [17] (**LI**, *n* = 25) and sTfR/logFerr < 1.5 for normal iron status (**NI**, *n* = 25) and the corresponding infant samples were then analysed at all 5 time points. There was a similar distribution of the 8 supplement groups among the **LI** and **NI** women (data not shown).

### 2.5 Statistical analysis & calculations

Statistical analysis was performed using DataDesk 6.3.1 (Data Description Inc., Ithaca, NY, USA) and all figures were drawn using GraphPad Prism 5.0 (GraphPad Software, San Diego CA, USA). Data are reported as mean and standard deviation (SD) for normally distributed or geometric mean (+ 1SD, − 1SD) for negatively skewed data. Hierarchical linear models, adjusted for participant ID (identification code/study number) and age (and infant weight-for-age Z-score for infant biochemistry models only), were used to identify differences in variables over time. To identify overall group differences, the same hierarchical model was used with the addition of participant ID nested in group. To identify specific time point differences by group an interaction term between group and time point was included and Fisher's Least Significant Difference (LSD) post-hoc tests were reported. To determine the extent of within individual tracking over time, hierarchical models were used adjusting for age and participant ID and the F-ratio and P-value reported for each variable. Backward step-wise elimination was used to determine the predictors of plasma FGF23 concentration over time. Glomerular filtration rate (eGFR) was not a significant predictor of C-FGF23 concentration but was a significant predictor of I-FGF23. When included in the predictive models of C- and I-FGF23, eGFR made no material difference to the relationships between Hb, I- and C-FGF23 and so was not included in the models. There was a similar distribution of males (M) and females (F) in the **LI** and **NI** children (F/M: **LI** = 10/15, **NI** = 13/12) and there was no sex difference in any of the biochemical variables. There were sex differences in anthropometry but these were accounted for with the calculation of age and sex adjusted Z-scores and so sex was not included as a variable in the models. *P* < 0.05 was considered as statistically significant.

eGFR (mL/min) was estimated using the following equation $\frac{74.835}{{\mathrm{CysC}\;\left(\frac{\mathrm{mg}}{L}\right)}^{1.33}}$ [18]. Calcium was corrected for albumin (Ca-corr; mmol/L) by normalising to an albumin concentration of 36 g/L using a correction factor 0.0167 mmol Ca/g albumin [19]. This correction factor was calculated from the slope of the relationship between Ca and albumin using local reference data [7].

## 3 Results

### 3.1 Maternal factors during pregnancy

At 20 wk of pregnancy the mean (SD) age of the mothers was 29.4 (7.0) years of age and height was 1.61 (0.06) metres, neither of which differed significantly between the groups (*P* = 0.9 and *P* = 0.4 respectively). Mothers in the **LI** group tended to be lighter than the mothers in the **NI** group [53.0 (8.4) vs. 58.0 (11.8) kg, *P* = 0.1] and their body mass index lower [20.6 (2.7) vs. 22.1 (4.2) kg/m^2^, *P* = 0.1], though neither difference was statistically significant. The median (IQR) parity of the women was 3 (4); seven were on their first pregnancy. Number of pregnancies did not differ between groups (data not shown).

### 3.2 Maternal iron status during pregnancy

**Fig. 1:**
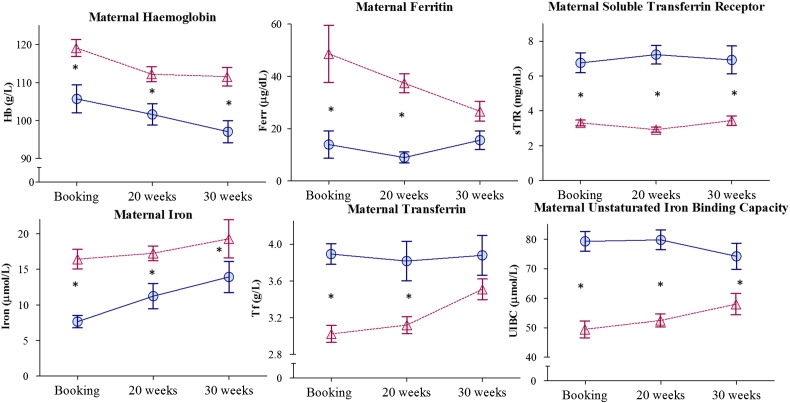
Maternal biochemistry. Mothers selected based on soluble transferring receptor (sTfR mg/L)/log ferritin (Ferr μg/L) index at 20 wk gestation: sTfR/log Ferr > 2.0 = Low Iron (blue circles), sTfR/log Ferr < 1.5 = Normal Iron (pink triangles). * indicates a significant difference between groups (P < 0.05) using least-squares post hoc tests as in Table 1.

Haemoglobin (Hb) decreased and unsaturated iron binding capacity (UIBC) and transferrin (Tf) increased significantly from booking to 30 wk of pregnancy when looking at all of the mothers together (Fig. 1 and Table 1.).

The **LI** mothers had significantly lower Hb, Ferr, Fe and significantly higher soluble sTfR, UIBC and Tf compared with the **NI** group throughout pregnancy (Fig. 1 and Table 1).

Forty-six percent of mothers had anaemia (Hb < 110 g/L) at booking [**LI**: 64% vs. **NI**: 28%, *P* = 0.01] which increased to 62% by 20 wk [**LI**: 72% vs. **NI**: 52%, *P* = 0.1] and to 64% by 30 wk [**LI**: 80% vs. **NI**: 46%, *P* = 0.01] (See Table 3). All markers of iron status tracked significantly within individual mothers throughout pregnancy (See Table 1) (i.e. mothers with low iron status remained low throughout gestation). The highest within individual tracking in iron markers over time was seen in sTfR and lowest in Fe.

### 3.3 Infant anthropometry from 12 to 104 wk of age

Weight-for-age, length-for-age, weight-for-length and head-circumference-for-age Z-scores decreased significantly from 12 to 104 wks. There were no group differences in length-for-age or head-circumference-for-age Z-scores at any time point whereas weight-for-age and weight-for-length Z-score were lower in the **LI** group compared to the **NI** group at 12 and 104 wk. (Table 2). There was no difference in gestational age at birth between groups [39.9 (1.04) vs. 39.9 (1.26) wk., *P* = 0.8].

### 3.4 Infant biochemistry from 12 to 104 wk of age

**Fig. 2:**
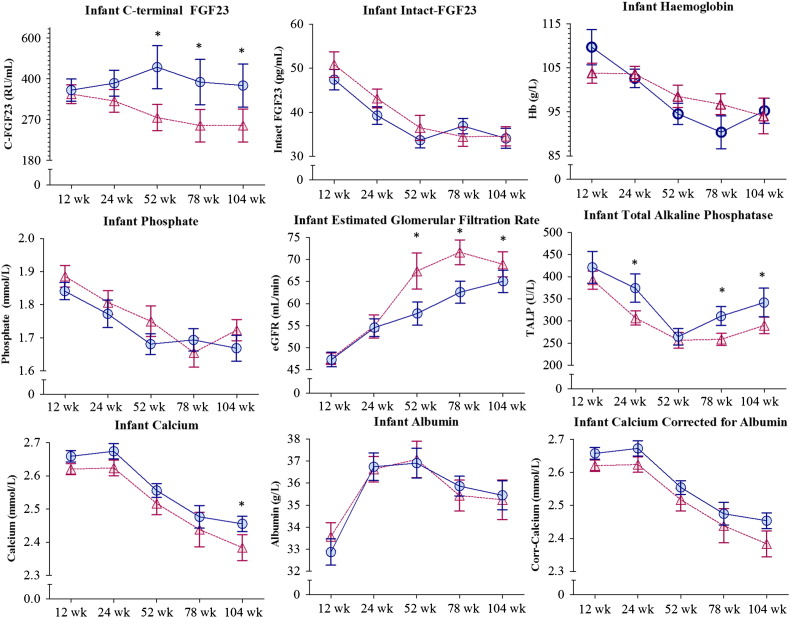
Infant biochemistry. Infants selected based on maternal soluble transferring receptor (sTfR mg/L)/log ferritin (Ferr μg/L) index at 20 wk gestation: sTfR/log Ferr > 2.0 = Low Iron (blue circles), sTfR/log Ferr < 1.5 = Normal Iron (pink triangles). * indicates a significant difference between groups (P < 0.05) using least-squares post hoc tests as in Table 4.

Circulating I-FGF23, Phos, Ca, TALP and Hb decreased and estimated glomerular filtration rate (eGFR) increased over time [overall mean (SD) I-FGF23: 49.1 (13.1) to 34.3 (10.9) pg/mL, Phos: 1.86 (0.14) to 1.68 (0.18) mmol/L, Ca: 2.63 (0.08) to 2.42 (0.16) mmol/L, TALP: 406 (133) to 318 (135) U/L, Hb: 107 (16) to 94 (16) g/L and eGFR: 59.9 (11.2) to 94.5 (23.4) mL/min all P ≤ 0.0001]. A drop was observed from 12 to 52 wk after which time point Hb and I-FGF23 plateaued from 52 to 104 wk (Fig. 2 and Table 4) whereas Ca remained elevated until 24 wk after which time point it dropped to 104 wk (Fig. 2 and Table 4). C-FGF23 did not change significantly with age [402 (218) to 487 (502) RU/mL, *P* = 0.6].

### 3.5 Infant iron status at 24 wk of age

Infant iron stores at 24 wk tended to be lower in the **LI** group compared to the **NI** group. This was particularly reflected by UIBC which was significantly higher in **LI** (P = 0.008) (Table 5.).

### 3.6 Maternal iron status and infant biochemistry

Infant circulating C-FGF23 concentration was significantly different by maternal iron status group (Fig. 2 and Table 4). C-FGF23 and TALP were higher in **LI** compared with **NI** from 52 wk for C-FGF23 and from 24 wk for TALP. eGFR was significantly lower in **LI** compared to **NI** from 52 wk and Ca was significantly lower in **NI** compared with **LI** at 104 wk only. All other infant biochemistry did not differ significantly by group (Fig. 2 and Table 4). Although C-FGF23 was elevated in the **LI** group compared to the **NI** group from 52 wk the group ∗ time point interaction term did not reach statistical significance (*P* = 0.08).

### 3.7 Circulating infant FGF23 and Hb concentrations

Seventy percent of infants had anaemia (Hb < 110 g/L) at 12 wk which increased over time to a peak of 97% at 78 wk (See Table 3) and this was not significantly different between group (Table 4).

After accounting for maternal group, C-FGF23 and infant Hb were negatively associated with each other at 24, 52, 78 and 104 wk with effect sizes ranging from β (S.E.) − 47.7 (9.9) %, r^2^ = 35.58%, P < 0.0001 at 52 wk to β (S.E.) − 33.4 (9.7) %, r^2^ = 25.3%, *P* = 0.001 RU/mL at 78 wk. Infant Hb was the strongest negative predictor of C-FGF23 concentration over time [β (S.E.) -20% (3%) RU/mL, P ≤ 0.0001 without adjustment and β (S.E.) − 21% (4%) RU/mL, P ≤ 0.0001 with adjustment for infant nutritional status (infant weight-for-age Z-score)]. Including infant Hb as a variable did not alter the magnitude of the group difference in C-FGF23 over time [β (S.E.) from 15.8 (8.2) %, *P* = 0.05 to β (S.E.) 15.3 (7.4) % *P* = 0.04 without adjustment and from 18.1 (8.4) %, *P* = 0.04 to β (S.E.) 17.6 (7.7) % *P* = 0.03 with adjustment for infant nutritional status].

Circulating I-FGF23 was not significantly associated with C-FGF23 or Hb at any time point [C-FGF23: − 0.4% (0.4%) RU/mL, *P* = 0.3; Hb: 0.003 (0.01) g/L, *P* = 0.8 and C-FGF23: − 0.5% (0.5%) RU/mL, *P* = *0.3*; Hb: 0.003 (0.01) g/L, *P* = *0.7* with adjustment for infant nutritional status]. Phos was the strongest positive predictor of I-FGF23 [β (S.E.) 31.4 (3.9) pg/mL, P ≤ 0.0001 and β (S.E.) 32.0 (3.9) pg/mL, P ≤ 0.0001] followed by eGFR [β (S.E.) − 0.07 pg/mL, P < 0.05 and β (S.E.) − 0.07 pg/mL, P < 0.05].

### 3.8 Tracking of infant anthropometry and biochemistry over time

All markers of anthropometry tracked significantly within individual infants across the first 2 years of life, particularly weight and weight-for-age Z-score (Table 2).

All biochemical markers corrected for weight-for-age Z-score, with the exception of Mg and Ca-corrected for albumin, tracked significantly within individual infants with time (Table 4). Among those infant biochemical markers with significant tracking, the highest was seen for TALP and C-FGF23.

## 4 Discussion

Neonatal iron deficiency is known to have adverse effects on various biological processes including neural development and cognitive performance [20]. Recently, murine studies have shown the importance of maternal iron status in pregnancy on offspring phosphate and vitamin D metabolism via regulatory changes in skeletal *FGF23* gene expression leading to increased circulating concentrations of FGF23 [6]. This present study is the first to investigate whether this is the case in humans; specifically to address whether there is an effect of maternal iron status during pregnancy on infant FGF23 and mineral metabolism over the first two years of life in a population with a high prevalence of iron deficiency anaemia. In addition, this is the first study to investigate the longitudinal changes in FGF23 (C-terminal and I-) in conjunction with mineral metabolites and glomerular filtration rate in full term infants through to childhood [21].

We showed that maternal Hb and markers of iron status track across pregnancy; women with low iron status continued to have low iron status until late gestation. In this sub-cohort of women selected by antenatal iron status, overall the anaemia rate in the mothers increased from 46% at booking to 64% by 30 wk of pregnancy despite supplementation. Similarly, the anaemia rate in the children increased from 70% of children at 12 wk to 90% at 2 years of age, and tracking of Hb concentration, as well as other biochemical and anthropometric markers, was seen across infancy. Individual tracking of variables was particularly strong for C-FGF23, TALP, weight-for-age and length-for-age Z-scores across infancy. As growth faltering is common during the first two years of life in rural Gambia [22] it was unsurprising that weight-for-age and length-for-age Z-scores in the **NI** group declined over time. However children in the **LI** group had even lower weight-for-age Z-scores compared to those in the **NI** group.

74% of all C-FGF23 concentrations measurements were well above the commonly accepted upper limit of normal of 125 RU/mL [8] and the extremely high measures of C-FGF23 (from 1000 to 3800 RU/mL) were seen only in children with anaemia. C-FGF23 concentration did not change significantly with age throughout the first 2 years of life in these children. The main finding from this study is that C-FGF23 was higher in the children born from mothers with a low sTfR/Ferr index (**LI**) diverging sometime between 6 and 12 months of life and lasting until at least 2 years of age compared to those in the **NI** group. In addition TALP was also higher in **LI** from 24 wk of age compared to the **NI** group. TALP when elevated is a marker of metabolic bone disease and is elevated in children with rickets [23]. The increased TALP concentrations in the **LI** group may be indicating that poor iron status has a negative influence on bone health, possibly mediated through its association with elevated FGF23 production.

Interestingly, I-FGF23 and Phos followed similar trajectories to each other throughout infancy; a sharp decrease over the first year and then a plateau during the 2nd year while eGFR in the **NI** group had an opposite trajectory; a sharp increase over the first year and then a plateau during the 2nd year. I-FGF23, rather than eGFR, was the strongest predictor of Phos over time, after accounting for maternal iron status. It is unknown whether circulating FGF23 changes in women during pregnancy although it has been shown to increase in pregnant mice [24]. Additional mice studies have demonstrated that the foetus produces its own FGF23 and that circulating maternal FGF23 does not cross the placenta [25]. Furthermore, as the half-life of FGF23 is less than an hour [26] the infant circulating FGF23 concentrations measured in our study reflects infant rather than maternally derived FGF23.

Our study provides evidence in support of the finding of Clinkenbeard et al., in mice of *in utero* effects of iron on FGF23 regulation [6]. The Clinkenbeard study involved feeding pregnant mice a normal or low iron diet from 14 days of pregnancy through to weaning (21 days after birth). Their study showed cross-sectional differences at 21 days of life in the offspring of the iron-depleted dams; including a higher C-FGF23 and I-FGF23 and lower Phos concentration compared with offspring of the iron-replete dams. Whether or not these group differences were present before or after 21 days of life was not investigated. In our study in humans we detected group differences in C-FGF23, but not in I-FGF23 or Phos, depending on maternal iron status in pregnancy which appeared from around 6 months of life and remained to at least 2 years of age. This fits in with the timing of cessation of exclusive breastfeeding which occurred at a mean age of 5.4 months in these children which did not differ significantly between groups. The findings of similar concentrations of C-FGF23 in the two groups for the first 6 months of life may suggest that both groups of infants were born with sufficient iron stores for the first 6 months of life. Thereafter, those infants born to mothers with low iron status may more rapidly become iron depleted. Alternatively, the **LI** children may have been exposed to a similar iron-poor environment as their mothers after the introduction of solid foods (~ 6 months of age) compared to the **NI** group. However, this is a less likely explanation as the markers of iron status suggest that the **LI** children were showing signs of poor iron status by 6 months of age.

There are two commercial assays available to measure human FGF23 in blood. The intact assay detects epitopes which cross the cleavage site of FGF23 and therefore detects only the intact protein whereas the C-terminal assay detects epitopes within the C-terminal of the protein and thus detects both the intact protein and cleaved C-terminal fragments. The intact protein is thought to be phosphaturic whereas there is conflicting evidence with regards to the biological activity of the C- and N- terminal fragments [27], [28].

The pathway by which low iron increases C-FGF23 expression is not clear but growing evidence points towards an involvement of the hypoxia-inducible-factor (HIF) pathway, activated by anaemia and hypoxia, in the increase in *FGF23* gene expression [5], [6]. This results in an increase of production and of intra- and extracellular degradations of the FGF23 hormone leading to increases in circulating C-FGF23 concentration but unchanged or slightly elevated concentrations of I-FGF23. Whether or not there are additional direct effects of iron on the downstream processing and catabolism of FGF23 protein is unclear. If tissue hypoxia is the driver of osteocyte *FGF23* gene expression rather than iron deficiency per se these findings may have implications for a number of disease states of chronic hypoxia such as cystic fibrosis (CF) and chronic pulmonary disorder (COPD) as well as complications during childbirth. Both CF and COPD patients are known to have compromised bone health and a study in COPD patients has shown that Phos and FGF23 metabolism is altered [29]. Elevation of *FGF23* gene expression as a result of chronic hypoxia is a plausible explanation of these findings.

An additional finding was the consistently and significantly lower infant eGFR in children in the **LI** group compared to the **NI** group appearing after 24 wk of age suggesting a potential role of iron in neonatal renal development. In support of this possibility, a study in rats showed that iron deficient neonatal rats had abnormal renal development including lower glomerular density, area and count, despite similar growth rates to iron replete neonates [30].

Limitations of the study include that the mothers and infants were participants of a supplementation trial [15]. Although all women received the same amount of iron and folic acid throughout pregnancy (60 mg of Fe and 400 μg of folic acid daily) and there was an equal distribution of all supplement groups between **LI** and **NI,** no account could be made of possible supplement effects. In addition, more frequent sampling in the children and additional markers of iron status, hypoxia and markers of vitamin D metabolism would have provided further mechanistic insights. We can assume from previous studies in this population that women and children in this present study had good vitamin D status, as determined by 25-hydroxyvitamin D concentration [31], [32]. However, we do not know whether or not FGF23-driven group differences in 1,25-dihydroxyvitamin D were present because they were not measured in this study. The tendency towards a lower BMI in **LI** mothers compared with the **NI** mothers raises the possibility that poor overall nutrition during pregnancy rather than specifically iron deficiency may be responsible for the FGF23 effects in infancy. Only a placebo-controlled iron supplementation trial during pregnancy would be able to answer this question definitely.

In conclusion, this study suggests that poor maternal iron status leads to higher infant C-FGF23 and TALP concentrations in human infants and young children but had no significant effect on infant I-FGF23. These findings further highlight the likely public health importance of preventing iron deficiency during pregnancy. Whether or not children who are born to iron deficient mothers have persistently high concentrations of these metabolites and are more likely to be at risk of impaired bone development and pre-disposed to rickets requires further research.

## Funding

This research was jointly funded by the MRC and the Department for International Development (DFID) under the MRC/DFID Concordat agreement: programme numbers U105960371, U123261351 and MC-A760-5QX00.

## Authors' roles

Study design: VSB, AP, MKD, AMP and SEM. Study conduct: VSB. Data analysis: VSB and AP. Data interpretation: VSB, AP and SEM. Drafting manuscript: VSB. Approving final version of manuscript: VSB, AP, MKD, AMP and SEM. VSB takes responsibility for the integrity of the data analysis. ENID Trial design: SEM, MKD and AMP.

## Conflicts of interest

All authors state that they have no conflicts of interest.

## Figures and Tables

**Table 1:** Maternal markers of iron status.

Maternal factors	Week	Mothers with low iron	Mothers with normal iron	Group difference P-value		Overall change with time		Within-individual tracking	
(*n* = 25)	(*n* = 25)	Time point	Overall	β (S.E.) years	P-value	F-ratio	P-value
Haemoglobin (g/L)	Bk	107 (18)	118 (12)	**0.0003**	**0.0006**	− 25.9 (7.1)	**0.0004**	4.2	**0.0001**
20	102 (14)	110 (9)	**0.008**
30	98 (15)	112 (13)	**0.0001**
Ferritin* (μg/L)	Bk	8.7 (3.6, 21.1)	35.8 (10.8, 118.5)	**0.0002**	**0.0001**	− 0.5 (0.5)	0.3	3.9	**0.0001**
20	6.6 (3.0, 14.4)	33.6 (20.1, 56.1)	**0.0003**
30	14.5 (8.0, 26.3)	20.9 (11.0, 39.7)	0.1
Iron (μmol/L)	Bk	7.6 (4.3)	16.4 (6.7)	**0.0003**	**0.0001**	9.0 (6.0)	0.1	1.7	**0.02**
20	11.2 (8.6)	17.2 (5.2)	**0.01**
30	13.9 (10.4)	19.3 (12.8)	**0.02**
sTfR* (mg/L)	Bk	5.9 (3.9, 9.1)	3.2 (2.5, 4.1)	**0.0001**	**0.0001**	0.23 (0.19)	0.2	8.7	**0.0001**
20	6.7 (4.8, 9.5)	2.9 (2.1, 3.9)	**0.0001**
30	6.0 (3.6, 9.9)	3.5 (2.5, 4.7)	**0.0001**
UIBC^a^ (μmol/L)	Bk	79.2 (16.0)	49.1 (13.9)	**0.0001**	**0.0001**	19.2 (9.9)	**0.06**	4.2	**0.0001**
20	79.7 (15.9)	52.1 (10.7)	**0.0001**
30	74.1 (20.3)	57.6 (17.6)	**0.0001**
Transferrin (g/L)	Bk	3.8 (0.5)	3.0 (0.4)	**0.0001**	**0.0001**	1.11 (0.44)	**0.01**	2.4	**0.0004**
20	3.8 (0.9)	3.1 (0.4)	**0.0001**
30	3.8 (0.9)	3.5 (0.5)	0.07

Group defined by maternal soluble transferring receptor (sTfR mg/L)/log ferritin (Ferr μg/L) index at 20 wk of gestation: sTfR/log Ferr > 2.0 = Low Iron, sTfR/log Ferr < 1.5 = Normal Iron. Data are mean (SD) for normally distributed data and geometric mean (− 1SD, + 1SD) for skewed variables*. Overall group difference was determined using hierarchical models with the independent variable adjusted for time point, group and ID (ID nested in group). Time point difference between group was determined as for the overall group difference with the addition of a group ∗ time point interaction term where post-hoc tests were used to determine group differences at specific time points. Overall change with time was calculated using hierarchical model with the independent variable adjusted for ID and age (years) and reported as the Β-coefficient (S.E.) in years for normally distributed independent variables and as a percentage for skewed variables*. Within-individual tracking was determined with hierarchical models adjusting for age (years) and ID and the ID F-ratio and P-value are reported.

a UIBC is unbound iron binding capacity.

**Table 2:** Infant anthropometry.

Infant factor	Week	Low maternal iron	Normal maternal iron	Group difference P-value		Overall change with time		Within-individual Tracking	
(*n* = 25)	(*n* = 25)	Time point	Overall	β (S.E.) yrs	P-value	F-ratio	P-value
Age (y)	12	0.25 (0.15)	0.25 (0.01)	0.8	0.4	NA	NA	NA	NA
24	0.47 (0.02)	0.46 (0.02)	0.2
52	1.00 (0.01)	1.00 (0.01)	0.7
78	1.51 (0.02)	1.50 (0.01)	0.6
104	2.00 (0.01)	2.00 (0.01)	0.7
Weight-for-height Z-score	12	− 0.92 (1.50)	− 0.53 (1.11)	**0.06**	0.5	− 0.14 (0.07)	**0.06**	8.3	**0.0001**
24	− 0.71 (0.98)	− 0.67 (1.31)	0.8
52	− 0.99 (1.02)	− 0.99 (1.40)	0.9
78	− 0.95 (0.73)	− 0.83 (1.36)	0.7
104	− 1.18 (0.78)	− 0.69 (1.36)	**0.02**
Length-for-age Z-score	12	− 0.44 (1.38)	− 0.16 (1.47)	0.2	0.3	− 0.69 (0.07)	**0.0001**	9.2	**0.0001**
24	− 0.44 (0.92)	− 0.26 (1.25)	0.3
52	− 1.12 (1.05)	− 0.88 (1.02)	0.2
78	− 1.35 (0.95)	− 1.03 (1.06)	0.09
104	− 1.55 (0.79)	− 1.35 (1.21)	0.3
Weight-for-age Z-score	12	− 1.06 (0.89)	− 0.58 (1.17)	**0.001**	0.3	− 0.36 (0.05)	**0.0001**	17.3	**0.0001**
24	− 0.86 (1.03)	− 0.73 (1.23)	0.4
52	− 1.28 (1.03)	− 1.15 (1.29)	0.4
78	− 1.34 (0.85)	− 1.08 (1.18)	0.08
104	− 1.67 (0.82)	− 1.19 (1.15)	**0.001**
Head-circumference-for-age-Z-score	12	− 0.50 (0.87)	− 0.27 (1.08)	0.9	0.8	− 0.40 (0.07)	**0.0001**	9.5	**0.0001**
24	− 0.48 (0.90)	− 0.53 (1.13)	0.9
52	− 0.77 (0.97)	− 1.07 (1.36)	0.7
78	− 1.02 (1.75)	− 1.03 (1.32)	1
104	− 0.97 (1.02)	− 1.15 (1.32)	0.9

Infants selected based on maternal soluble transferring receptor (sTfR mg/L)/log ferritin (Ferr μg/L) index at 20 wk gestation: sTfR/log Ferr > 2.0 = Low Iron, sTfR/log Ferr < 1.5 = Normal Iron. Data are mean (SD). Overall group difference was determined using hierarchical models with the independent variable adjusted for time point, group and ID (ID nested in group). Time point difference between group was determined as for the overall group difference with the addition of a group ∗ time point interaction term where post-hoc tests were used to determine group differences at specific time points. Overall change with time was calculated using hierarchical model with the independent variable adjusted for ID and age (years) and reported as the Β-coefficient (S.E.) in years. Within-individual tracking was determined with hierarchical models adjusting for age (years) and ID and the ID F-ratio and P-value are reported. “WHO Anthro” was used to determine Z-scores [16].

**Table 3:** Anaemia in pregnancy and infancy.

Time point	% Anaemia haemoglobin < 110 g/L			Chi square P-value
All	Low maternal iron	Normal maternal iron
*Mother*				
Booking	46.0	64.0	28.0	0.01
20 weeks	62.0	72.0	52.0	0.1
30 weeks	63.8	80.0	45.5	0.01

*Infant*				
12 weeks	70.5	70.0	70.8	0.9
24 weeks	76.6	78.3	75.0	0.8
52 weeks	88.1	90.9	85.0	0.6
78 weeks	97.3	100.0	94.7	0.3
104 weeks	89.7	90.0	89.5	0.9

Percentage of mothers and infants with anaemia defined by the World Health Organisation cut-off [33] at each time point split by maternal soluble transferring receptor (sTfR mg/L)/log ferritin (Ferr μg/L)index at 20 wk gestation: sTfR/log Ferr > 2.0 = Low Iron, sTfR/log Ferr < 1.5 = Normal Iron.

**Table 4:** Infant biochemistry.

Factor	Week	Low maternal iron	Normal maternal iron	Group difference P-value		Overall change with time		Within-individual tracking	
(*n* = 25)	(*n* = 25)	Time point	Overall	Β (S.E.) yrs	P-value	F-ratio	P-value
Haemoglobin (g/L)	12	109 (18)	104 (11)	0.06	0.9	− 0.6 (0.1)	**0.0001**	2.4	**0.0001**
24	103 (10)	104 (08)	0.8
52	95 (11)	99 (11)	0.3
78	90 (16)	97 (10)	0.07
104	95 (13)	94 (18)	0.6
Intact-fibroblast growth factor-23 (pg/mL)	12	47.4 (11.6)	50.8 (14.5)	0.2	0.5	− 6.9 (1.0)	**0.0001**	4.2	**0.0001**
24	39.2 (10.2)	43.1 (10.7)	0.1
52	33.6 (8.9)	36.5 (14.2)	0.2
78	36.8 (8.4)	34.5 (10.9)	0.3
104	34.0 (11.3)	34.5 (10.7)	0.9
C-terminal fibroblast growth factor-23 (RU/mL)*	12	365 (212, 629)	347 (220, 548)	0.5	**0.04**	− 3.2 (6.6)	0.6	4.8	**0.0001**
24	391 (207, 739)	325 (187, 563)	0.2
52	457 (160, 1307)	276 (145, 525)	**0.003**
78	395 (131, 1197)	255 (115, 564)	**0.006**
104	382 (133, 1101)	255 (114, 570)	**0.008**
Calcium (mmol/L)	12	2.66 (0.08)	2.62 (0.08)	0.3	0.1	− 0.1 (0.02)	**0.0001**	4.5	**0.04**
24	2.67 (0.11)	2.62 (0.12)	0.2
52	2.55(0.09)	2.52 (0.16)	0.4
78	2.47 (0.17)	2.43 (0.25)	0.3
104	2.45 (0.11)	2.38 (0.21)	**0.04**
Phosphate (mmol/L)	12	1.84 (0.12)	1.88 (0.15)	0.6	0.4	− 0.1 (0.02)	**0.00001**	2.3	**0.0001**
24	1.77 (0.20)	1.80 (0.17)	0.5
52	1.68 (0.14)	1.75 (0.22)	0.5
78	1.69 (0.17)	1.65 (0.22)	0.8
104	1.66 (0.19)	1.72 (0.16)	0.1
Magnesium (mmol/L)	12	0.86 (0.06)	0.87 (0.05)	0.9	0.8	− 0.03 (0.01)	**0.02**	1.0	0.41
24	0.88 (0.05)	0.87 (0.12)	0.7
52	0.87 (0.05)	0.87 (0.09)	0.8
78	0.84 (0.13)	0.84 (0.12)	0.9
104	0.85 (0.07)	0.83 (0.12)	0.7
Total alkaline phosphatase (U/L)	12	420.8 (165.9)	392.4 (93.6)	0.2	0.1	− 31.3 (9.6)	**0.001**	6.4	**0.0001**
24	373.6 (153.9)	306.8 (77.9)	**0.001**
52	263.7 (81.3)	256.7 (83.3)	0.6
78	310.2 (106.7)	258.7 (66.9)	**0.002**
104	340.9 (162.0)	289.9 (93.7)	**0.001**
Cystatin C (mg/L)	12	1.22 (0.24)	1.19 (0.14)	0.4	0.1	− 0.17 (0.02)	**0.0001**	4.0	**0.0001**
24	1.05 (0.16)	1.07 (0.22)	0.9
52	1.00 (0.18)	0.88 (0.22)	**0.008**
78	0.93 (0.17)	0.81 (0.16)	**0.003**
104	0.89 (0.16)	0.84 (0.16)	0.1
Estimated glomerular filtration rate (mL/min)	12	59.3 (12.8)	59.8 (10.1)	0.5	**0.03**	18.3 (2.2)	**0.0001**	4.0	**0.0001**
24	72.0 (17.5)	73.8 (24.2)	0.4
52	78.5 (21.0)	89.2 (22.8)	**0.005**
78	89.3 (25.7)	101.1 (23.3)	**0.006**
104	93.5 (26.8)	97.0 (20.7)	**0.04**
Albumin (g/L)	12	32.85 (2.72)	33.56 (2.93)	0.8	0.6	0.9 (0.3)	**0.009**	2.6	**0.0001**
24	36.72 (2.69)	36.62 (2.81)	0.8
52	36.88 (2.95)	37.06 (3.85)	0.9
78	35.84 (2.23)	35.43 (3.51)	0.3
104	35.43 (3.31)	35.24 (4.49)	0.3
Calcium-corrected for albumin (mmol/L)	12	2.71 (0.07)	2.66 (0.06)	0.2	**0.01**	− 0.15 (0.01)	**0.0001**	1.2	0.2
24	2.66 (0.09)	2.61 (0.11)	0.1
52	2.54 (0.06)	2.49 (0.15)	0.3
78	2.47 (0.16)	2.44 (0.21)	0.4
104	2.46 (0.09)	2.39 (0.16)	0.06

Infants selected based on maternal soluble transferring receptor (sTfR mg/L)/log ferritin (Ferr μg/L) index at 20 wk gestation: sTfR/log Ferr > 2.0 = Low Iron, sTfR/log Ferr < 1.5 = Normal Iron. Data are mean (SD) for normally distributed data and geometric mean (− 1SD, + 1SD) for skewed variables*. Overall group difference was determined using hierarchical models with the independent variable adjusted for time point, infant weight-for-age Z-score, group and ID (ID nested in group). Time point difference between group was determined as for the overall group difference with the addition of a group ∗ time point interaction term where post-hoc tests were used to determine group differences at specific time points. Overall change with time was calculated using hierarchical model with the independent variable adjusted for infant weight-for-age Z-score, ID and age (years) and reported as the Β-coefficient (S.E.) in years for normally distributed independent variables and as a percentage for skewed variables*. Within-individual tracking was determined with hierarchical models adjusting for age (years), infant weight-for-age Z-score and ID and the ID F-ratio and P-value are reported.

**Table 5:** Infant iron status markers at 24 wk of age.

Analyte at 24 weeks	All	Low maternal iron	Normal maternal iron	P-value
Haemoglobin (g/L)	103 (9)	103 (10)	104 (9)	0.7
Ferritin*(μg/L)	26.7(8.7, 81.3)	12.9 (6.9, 69.1)	32.2 (11.1, 94.0)	0.2
Iron (μmol/L)	6.3 (2.3)	5.9 (2.4)	6.7 (2.1)	0.2
Transferrin (g/L)	2.9 (0.6)	3.1 (0.5)	2.9 (0.6)	0.1
Unbound iron binding capacity (μmol/L)	62.4 (15.3)	68.3 (15.8)	56.8 (12.5)	0.008

⁎ Data are mean (SD) for normally distributed data and geometric mean (− 1SD, + 1SD) for skewed variables.
